# The Mutant Mouse Resource and Research Center (MMRRC): the NIH-supported National Public Repository and Distribution Archive of Mutant Mouse Models in the USA

**DOI:** 10.1007/s00335-021-09894-0

**Published:** 2021-07-27

**Authors:** James Amos-Landgraf, Craig Franklin, Virginia Godfrey, Franziska Grieder, Kristin Grimsrud, Ian Korf, Cat Lutz, Terry Magnuson, Oleg Mirochnitchenko, Samit Patel, Laura Reinholdt, K. C. Kent Lloyd

**Affiliations:** 1 Department of Veterinary Pathobiology, College of Veterinary Medicine, University of Missouri, Columbia, MO USA; 2 Department of Genetics and Office of the Vice Chancellor for Research, Univeristy of North Carolina-Chapel Hill, Chapel Hill, NC USA; 3 Division of Comparative Medicine, Office of Research Infrastructure Programs, Division of Program Coordination, Planning, and Strategic Initiatives, Office of the Director, National Institutes of Health, Bethesda, MD USA; 4 Mouse Biology Program, University of California, Davis, CA USA; 5 Department of Molecular and Cellular Biology, College of Biological Sciences and Bioinformatics Core, Genome Center, University of California, Davis, CA USA; 6 The Jackson Laboratory, Bar Harbor, ME USA

## Abstract

The Mutant Mouse Resource and Research Center (MMRRC) Program is the pre-eminent public national mutant mouse repository and distribution archive in the USA, serving as a national resource of mutant mice available to the global scientific community for biomedical research. Established more than two decades ago with grants from the National Institutes of Health (NIH), the MMRRC Program supports a Consortium of regionally distributed and dedicated vivaria, laboratories, and offices (Centers) and an Informatics Coordination and Service Center (ICSC) at three academic teaching and research universities and one non-profit genetic research institution. The MMRRC Program accepts the submission of unique, scientifically rigorous, and experimentally valuable genetically altered and other mouse models donated by academic and commercial scientists and organizations for deposition, maintenance, preservation, and dissemination to scientists upon request. The four Centers maintain an archive of nearly 60,000 mutant alleles as live mice, frozen germplasm, and/or embryonic stem (ES) cells. Since its inception, the Centers have fulfilled 13,184 orders for mutant mouse models from 9591 scientists at 6626 institutions around the globe. Centers also provide numerous services that facilitate using mutant mouse models obtained from the MMRRC, including genetic assays, microbiome analysis, analytical phenotyping and pathology, cryorecovery, mouse husbandry, infectious disease surveillance and diagnosis, and disease modeling. The ICSC coordinates activities between the Centers, manages the website (mmrrc.org) and online catalog, and conducts communication, outreach, and education to the research community. Centers preserve, secure, and protect mutant mouse lines in perpetuity, promote rigor and reproducibility in scientific experiments using mice, provide experiential training and consultation in the responsible use of mice in research, and pursue cutting edge technologies to advance biomedical studies using mice to improve human health. Researchers benefit from an expansive list of well-defined mouse models of disease that meet the highest standards of rigor and reproducibility, while donating investigators benefit by having their mouse lines preserved, protected, and distributed in compliance with NIH policies.

## Accelerating the use of mutant mice in biomedical research

Beginning in the early 1990's, a nearly three decade expansion of the repertoire of mouse models that could be used experimentally for the study of human diseases began (Davisson and Taft 2006; Gurumurthy and Lloyd 2019). Sequential emergence and experimental application of a myriad of technologies, including ethylnitrosurea (ENU) mutagenesis, homologous recombination in embryonic stem (ES) cells, transgenic, conditional targeting (e.g., Cre), transposons, and programmable endonucleases (e.g., CRISPR/Cas9) all contributed to a rapid growth in the design, development, and production of a variety of mutant mice for biomedical research. Eventually, the few commercial and not-for-profit mouse distributors that were archiving and maintaining these new mouse lines were becoming overwhelmed, precipitating risky and capricious trafficking of mice among individual investigator laboratories, often with naive disregard of quality control standards, genetic fidelity, contamination with adventitious pathogens, or the spread of transmissible infections. As a consequence, many mouse lines were unavailable or if available unaccessible, and those that could be obtained were often unreliable due to poorly characterized and often erroneous genotype, insufficient documentation, or poor health. In turn, results published using these models failed replication, preclinical research using animals became unpredictable and irreproducible, and scientific progress was being hampered. These and other challenges were spreading widely, and the reliability and reproducibility of scientific results using mice (and other animals) was being seriously questioned, especially in translational preclinical studies (Kilkenny 2009).

## A trans-national network of non-profit mouse repositories

In response to these mounting pressures, the US National Institutes of Health (NIH) in 1998 convened a panel of internationally renowned scientists to draft recommendations for facilitating broad but controlled access to mouse resources under rigorous quality control standards (Battey 1999). The top recommendation of this group was for the federal government to create a consortium of archive and distribution repositories to solicit, accept, import, validate, maintain, preserve, and disseminate mutant mouse lines, especially those generated by NIH-funded researchers and laboratories. Although expected to have the scientific expertise, experience, capability, and capacity to manage mice and mouse resources, NIH-supported repositories were first and foremost tasked with serving as an unrestricted resource of the burgeoning mutant mouse lines for experimental use by the academic research community. Second, they were mandated to contribute to science and technology to enhance and develop their holdings, such as research into embryo cryopreservation and archiving frozen germplasm. Third, repositories were to serve the broad research community in diverse disease areas, and not focus on a particular category of mouse resource or disease area. Fourth, they were to make mice available to the research community on a local, regional, and national (and eventually international) basis, irrespective of where the repositories were located geographically. Finally, funded repositories were expected to have robust animal infrastructure (vivaria, technicians, veterinary and laboratory support), mouse husbandry and care experience, and infectious and genetic quality control and assurance standards and policies in place to ensure research reproducibility and integrity. These characteristics have since become fundamental expectations of centralized mouse repository systems worldwide (Donahue 2012).

## Birth of the MMRRC

What arose from these discussions was the creation by NIH of an extramural Mutant Mouse Regional Resource Center (MMRRC) Program which was intended to fund competitive applications from public and private entities committed to accepting, maintaining, and disseminating mutant mouse models to the biomedical research community (Grieder 2002). The first Request for Applications (RFA-RR-99-001) was issued by the NIH National Center for Research Resources (NCRR; now under the authority of the NIH Office of Research Infrastructure Programs [ORIP], Division of Program Coordination, Planning, and Strategic Initiatives [DPCPSI], Office of the Director) which officially established the MMRRC Program as the nation’s premier publicly accessible mutant mouse archive and distribution repository system. The MMRRC was rechristened in 2014 as the Mutant Mouse *Resource and Research* Center to reflect the expansion of its mission to include resource-related research that is intended to promote greater effectiveness and efficiencies in service to the biomedical research community without bias for a particular category of mouse model or research. In practical terms, the MMRRC Program provided funding to host institutions willing to participate in a national Consortium of individual, regionally distributed Centers with dedicated infrastructure (including barrier housing and microbial quality control), cryopreservation and reconstitution capabilities, proven standard operating procedures (SOPs) and surveillance protocols for infectious pathogens and environmental contaminants, and sufficient professional, scientific, and technical expertise in quality control and other activities to maintain the genetic integrity of mice and germplasm at first, then later murine embryonic stem (ES) and hybridoma cell lines.

The first NIH awards establishing archive and distribution Centers were made in 1999, two to academic institutions (UC Davis and UNC-Chapel Hill) and two to commercial/academic partnerships (Harlan/University of Missouri-Columbia and Taconic/SUNY-Albany) which together with a separately funded Informatics, Coordination and Service Center (ICSC) collectively became known as the MMRRC Consortium. At the first competitive renewal in 2005, the two commercial partners had left the MMRRC Consortium while funding was awarded to the University of Missouri-Columbia to operate alone as the third Center. In response to the growth and success of the three academic-based Centers during the first 10 years of funding, at the 2nd competitive renewal in 2010 The Jackson Laboratory was added as the fourth Center. All four Centers then successfully renewed in 2015 and again in 2020. Today, each Center exists in a research-intensive environment that synergizes with in-house genetic and programmatic resources and expertise that reinforces the MMRRC Program mission (Table 1) and engages in close coordination and working relationship between Centers.

**Table 1:** Location and web addresses of (current) Centers in the MMRRC Consortium

Host Institution (year established)	Web Address
University of Missouri, Columbia (1999*)	mmrrc.missouri.edu
University of California, Davis (1999)	mmrrc.ucdavis.edu
The Jackson Laboratory (2010)	jax.org/mmrrc
University of North Carolina, Chapel Hill (1999)	med.unc.edu/mmrrc

*Initially in partnership with Harlan, then established independently 2005

The original mandate of the Centers participating in the MMRRC Consortium was to accept, maintain, and distribute mutant mice to the scientific community. This approach would ensure the viability, genetic identity and background, pathogen-free status, and long-term availability of valuable mutant mouse models for research. The four Centers operate under a common umbrella of SOPs that define best practices for the operation of a twenty-first century mouse archive and distribution repository system (Table 2).

**Table 2:** MMRRC consortium best practices

All MMRRCs observe best practices in the following activities:
Importation, rederivation, maintenance (e.g., husbandry, care, and welfare), and distribution
Cryopreservation and recovery, pathogen surveillance and health monitoring, mouse genetics
Information technology, database management, and website and online services
Communications, outreach, and education

Centers harmonized internal SOPs within the MMRRC Consortium while maximizing individual efficiencies. The Centers began serving as *centers of excellence* in mouse biology and phenotyping, thereby facilitating and extending the validity, reproducibility, and scientific value of these models. In some cases they have also earned “approved vendor” status from institutions of requesting investigators, facilitating direct import of mice into research colonies which bypasses costly delays associated with importation quarantines and rederivation.

## Opening its doors for business

When launched, Centers within the MMRRC Consortium began accepting applications from investigators to submit their mutant mouse lines for archiving and distribution. It was at this time that the variety and types of mouse lines…from those derived from ethylnitrosurea (ENU) injection into male mice, random gene and promoter trap experiments in murine ES cells, mice expressing specific recombination enzymes (CRE, FLP), to a host of new transgenic and knockout mice. Within a year, the MMRRC Program was adapting to this changing environment and broadening opportunities for acceptance of a wider variety of mouse lines by establishing three types of submissions (Table 3). Most submissions were categorized as Type 1 in which a mutant mouse line was developed and characterized by the donating investigator. Type 1 submission applications are reviewed monthly by video teleconference of a Coordinating Committee made up of the principal investigators of the Centers and ICSC and NIH program officials. These reviews are intended to determine whether an application for a Type 1 submission fulfills the following six criteria:

- Created and analyzed using sound scientific methods;
- Distinguishable genotypically and/or phenotypically from other publicly available lines;
- Exhibits scientifically rigorous and experimentally reproducible findings;
- Exists at no other public distribution repository;
- Available for distribution without restriction to academic researchers, and
- Controls are readily available or can be provided by the submitting investigator.

**Table 3:** Types of mouse submissions to the MMRRC Program

MMRRC donation type	Description
Type 1	Individual donating investigators
Type 2	NIH Program Officer-sanctioned mice/lines
Type 3	Contract to individual center

Although not required, a scientific publication or readily accessible phenotype data (e.g., via a web-based resource) describing the creation and study of the submitted mouse line adds considerable scientific data and documented evidence to facilitate review. Generally, a mouse line must meet all criteria to be accepted into the MMRRC. Upon review, MMRRC ICSC staff will work with the submitting investigator to obtain additional details or provide other options for depositing into the MMRRC (e.g., Type 3 donation) if their mouse line fails to meet acceptance criteria. The only cost to the submitting investigator is that incurred by shipping mice to the assigned Center. Other than that, all importation, rederivation, quality control testing, maintenance, and archiving costs are borne by NIH funds awarded to the host institution to support the Center. For example, once imported into the Center’s repository, tissue samples are taken for DNA extraction to confirm genotype of the mutant allele and genetic background, fecal pellets are harvested (or collected earlier by the submitting investigator before shipping mice) for microbiome analysis, mice are bred (if necessary) to generate live colonies, and sperm and/or embryos are cryoarchived. Mice are listed as available for distribution by browsing or searching the online catalog once fully curated and after confirmation of gene-specific genotype and genetic background, establishment of a cryopreserved archive and verification of viable cryorecovery, determination of pathogen-free status, and exclusion of unintended or contaminating DNA elements (e.g., Cre). Proceeds derived from modest fees charged to requesting investigators to obtain and ship mouse lines to requesting investigators provides program income to support distribution costs (e.g., cryorecovery supplies, technical effort, genotyping, holding costs, etc.) incurred by the Center.

Type 2 and Type 3 submissions undergo a similar level of rigorous assessment and review yet because of their origin or other special characteristics do not go through the same formal application process as Type 1 submissions. For example, the MMRRC Program provides a very limited number of Type 2 submission “slots” that are pre-selected by NIH categorical institutes for fast-track acceptance of mice from their funded scientists into the MMRRC. Type 3 submissions are primarily secured as large, thematic collections that were produced by large consortiums, commercial organizations, and/or private research entities. Some of these collections are submitted and made available for distribution as gene-targeted ES cells, several of which have been converted into mice and are available as frozen germplasm. As a result, over time the number of Type 3 mouse lines has significantly exceeded the number of either Type 1 or Type 2 mouse lines deposited into the MMRRC (Table 4)**.**

**Table 4:** Mutant mouse lines accepted into the MMRRC Program

Deposition type	Cumulative (2000–2020)
Type 1: Investigator initiated, MMRRC Program supported	1872
Type 2: NIH initiated, MMRRC Program supported	234
Type 3: Investigator initiated and supported	57,796
Totals	59,902

## Donation of mouse lines to the MMRRC

**Fig. 1:**
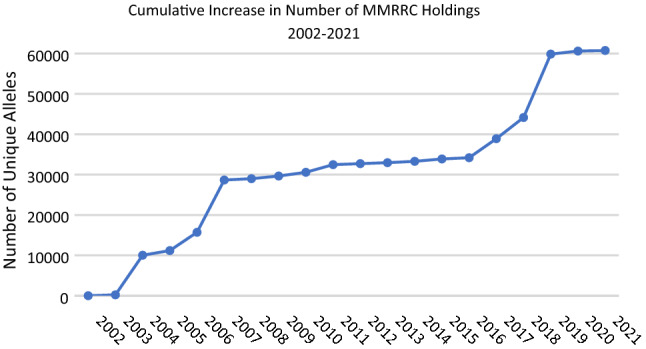
The cumulative annual increase in MMRRC Program holdings of unique mutant alleles and hybridoma cell lines beginning in 2002 to March 1, 2021. The change each year reflects the sum of all submissions (Type 1, Type 2, and Type 3) accepted and imported into the MMRRC repositories during that year

**Fig. 2:**
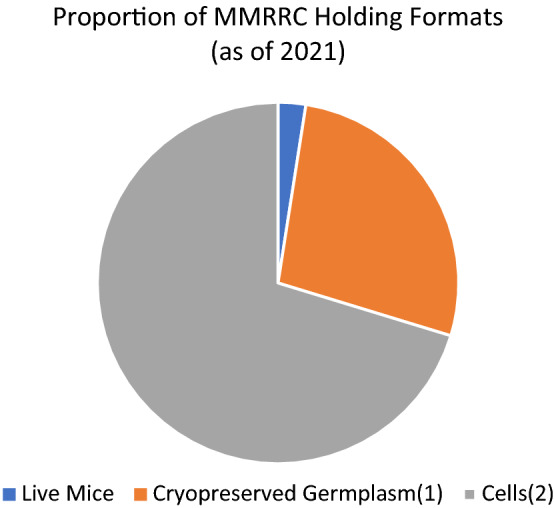
The proportional distribution of various formats (live, cryopreserved germplasm, cell lines) for all MMRRC holdings as of 2021. The number of mouse lines maintained as live breeding colonies is small relative to the germplasm and cell archives and reflects those lines undergoing active importation and awaiting cryopreservation and/or being actively distributed in response to orders for live mice; (1) a mutant allele maintained as a frozen embryo and/or sperm; (2) embryonic stem cells or hybridoma cell lines

The growth in number of holdings in the MMRRC Program has increased over the last 20 years to a total of 60,751 unique mutant alleles (Fig. 1), making it one of the largest public repositories of mutant mouse lines in the world today. These lines are available in one of more forms as live mice, frozen germplasm, and/or pluripotent ES cell lines or hybridomas (Fig. 2). These models are useful for research in many disease categories including oncology, cardiology, immunology, and neurology, developmental biology, and to study specific syndromes and diseases. Investigators interested in submitting their mouse line to the MMRRC Program can submit information online through the MMRRC website (https://www.mmrrc.org/submission/strain_submission_terms.php), contact the Strain Acquisition Coordinator at the ICSC, or contact the Import Coordinator at any of the Centers for more information, assistance, and instructions.

## Distribution of mouse lines from the MMRRC

**Fig. 3:**
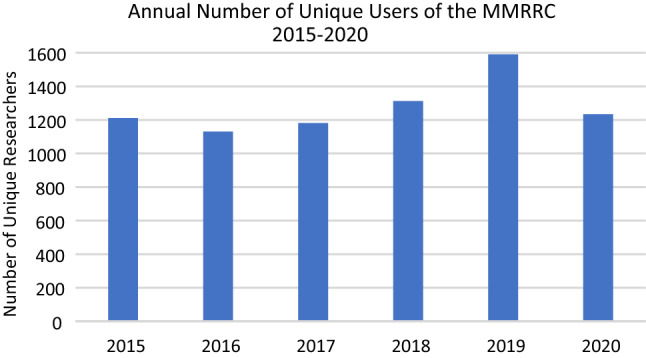
The number of unique researchers annually who ordered from the MMRRC repositories each year between 2015 and 2020. The sudden dropoff of orders during 2020 has been attributed to the effects of the temporary shutdown of non-essential research activity and shuttered research laboratories during the SARS-CoV-2 pandemic

**Fig. 4:**
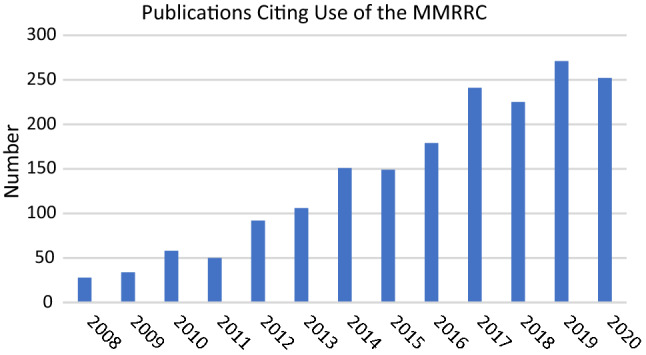
Annual number of peer-reviewed publications that cite or acknowledge ordering mice, germplasm, or cell lines from the MMRRC repositories between 2008 and 2020. As of March 1, 2021, a total of 1,924 publications have cited or acknowledge the use of the MMRRC. Unfortunately, because not all reseachers who publish remember to cite usage of the MMRRC, these numbers underrepresent the actual number of publications reporting on the use of mice, germplasm, or cell lines obtained from the MMRRC repositories

The number of researchers using the MMRRC Program and the number of orders for mutant mouse lines have steadily increased since the MMRRC Consortium started operations. For example, the number of unique users has averaged over 1276 per year over each of the last 5 years to a total of 16,303 by the end of 2020 (Fig. 3). Despite the recent and temporary drop in orders resulting from the impact of the global SARS-CoV-2 pandemic on research activity and onsite laboratory work during most of 2020, this number of users has translated into a substantial number of orders received and fulfilled overtime by the MMRRC since opening its doors (Table 5). Many researchers are returning customers who request not only additional mouse lines but also special services from a Center through their host institution. In addition, with the implementation of a system of unique Research Resource Identifiers (RRID; https://scicrunch.org/resources) (Bandrowski and Martone 2016) to search relevant databases (e.g., PubMed), at least 1924 research articles have been published to date using mouse lines obtained from the MMRRC Program (Fig. 4). This number of publications is surely an underrepresentation, as past publications often neglect to cite the MMRRC. New articles are discovered daily, reflecting the ongoing importance of the MMRRC Program as a vital public resource for research using mutant mice.

**Table 5:** Orders for mutant mouse lines from the MMRRC Program

Metric	Cumulative 2000–2020
Orders^1^	13,184
Investigators^2^	9591
Institutions^2^	6626

^1^Can include multiple mouse lines mice

^2^Unique

## Enhancing capacity to improve resource quality

As experts in mouse biology and related research, each Center also provides a number of ancillary services and procedures that can be accessed by users of the MMRRC. These services include colony management and breeding, assisted reproductive technologies, ES cell and microinjection, transgenic and other production (CRISPR/Cas9) approaches, genotyping and genetic analysis, in vivo and ex vivo phenotyping, clinical diagnostics, and anatomic pathology. Individuals, institutions or consortia may arrange for the acquisition, importation, cryopreservation and distribution of individual mouse lines or collections on a fee-for-service basis from a Center. A table of available services and contact information at each Center can be found at https://www.mmrrc.org/about/members.php

## Conducting resource-related scientific research

A critical component of the MMRRC Program is innovative research that enhances resource operations and services, such as developing optimal and more efficient means of cryopreservation, re-animation, genotyping, infectious disease control, and microbiome analysis. These efforts have included improvements in cryopreservation and recovery of C57BL/6J and C57BL/6N sperm and embryos (Li 2014; Mochida 2012), refinement of in vitro fertilization (IVF) and intracytoplasmic sperm injection (ICSI) procedures (Li 2015,2003), enhanced isolation, purification, and gene targeting in murine ES cells (Pettit 2009), development of high-density SNP genotyping panels (Mouse Universal Genotyping Arrays [MUGA]) to define genetic background and for speed congenics (Sigmon 2020), improved pathogen detection assays (Ericsson 2017), genome editing technologies (CRISPR/Cas9) (Modzelewski 2018) and the establishment of strategies by which to provide requesting investigators with mice on select complex microbiota (Hart 2018; Ericsson et al. 2017). In this way, the MMRRC Program is constantly adapting to the rapidly changing needs of the scientific community by mitigating negative impacts of growing mouse populations on the nation’s infrastructure. Further, electronic networking and innovative data management that enables integration of data and information from multiple sites, automated tools, curation, attention to cybersecurity, and quality control have been incorporated by the ICSC into the MMRRC Consortium. Centers provide training and professional expertise to maximize their impact and access by the scientific community.

## Coordination between centers in the MMRRC consortium

Centers and ICSC PIs in addition to meeting monthly convene with NIH program staff annually in person to discuss complex programmatic and strategic planning, allowing time to critically and objectively review and assess consortium-wide operational and performance metrics, discuss research goals and accomplishments, and develop strategies to address the evolving needs of the biomedical research community. Biennially these meetings are in association with the NIH-ORIP Resource Directors’ Meeting in Bethesda, Maryland. On alternate years, individual Centers host MMRRC Consortium meetings at their host institutions. An External Advisory Committee of five technical specialists and research scientists attend and provide critical review and valuable feedback on MMRRC Program performance and planning to which the MMRRC Consortium responds with action items and point-by-point reports. These individuals also serve to represent the scientific community and advise Center leadership on ways to enhance and improve the provision and delivery of services to its users. In addition, the MMRRC Consortium has established a number of internal working groups which facilitate collective discussions to address issues, challenges, and opportunities in management, administration, technology, intellectual property, research, and other areas that help the MMRRC Program continue to evolve and develop to effectively meet the needs of the research community.

## An emphasis on serving scientific needs

A primary goal of the MMRRC Program is to present a single, unified, online presence to best serve its customers. The ICSC coordinates customer service by maintaining a public web site (www.mmrrc.org) with access to a searchable repository catalog, an ordering and submission system, and centralized customer service support. Customer service staff at the ICSC are knowledgeable on all aspects of MMRRC Program services. Customers can communicate with customer service staff through dedicated MMRRC telephone numbers (800-910-2291 [North America], 530-757-5710 [international]), email addresses (service@mmrrc.org, support@mmrrc.org), and a service request tracking system. Standard customer operating procedures, including templates and institutional tracking of intellectual property issues, support both donors and purchasers and their affiliated research institutes as well as facilitate seamless coordination between all Centers in the MMRRC Consortium.

## Unified community outreach and education

The MMRRC promotes a unified identity to increase awareness of mouse resources within the research community. The MMRRC brand is used across all outreach materials, such as the MMRRC booth which ICSC staff take to scientific meetings, print and online advertisement, and pamphlets, buttons, and other collateral distributed at meetings and within outgoing shipments. The MMRRC Program is continuously developing new promotional methods to build awareness of its repository holdings and value-added services. Although together the Centers house a large number of mouse lines with a variety of phenotypes, usage depends completely on customer awareness. The ICSC profiles researchers based on their publications and correlates research interests with repository holdings. This information is then distributed through highly customized emails to inform researchers of specific and potentially useful mice and services for their research. The same system is used to solicit the submission of newly published mouse models. The immediate success of this system has been demonstrated by an increase in number of strain submissions, flow of traffic to the MMRRC web site, and volume of catalog searches. Comprehensive tools for assessing the impact of email campaigns are utilized to enhance their efficacy and to facilitate customer service support. Interested investigators can follow the MMRRC Program *@mmrrc*.

## Enagagment and interaction with complementary organizations

The MMRRC Program actively pursues opportunities to engage and interact with similar and complementary resources and entities. For example, the ICSC offers openly available, automatic updates of the mouse holdings at each Center. Popular subscribers include PubMed (https://pubmed.ncbi.nlm.nih.gov/), the Online Mendelian Inheritance of Man (OMIM, www.omim.org), the International Mouse Strain Resource (IMSR, http://www.findmice.org/), the Mouse Genome Informatics (MGI, http://www.informatics.jax.org/), and the International Mouse Phenotyping Consortium (IMPC, https://www.mousephenotype.org/). Together with the MMRRC these organizations represent a large share of commonly used entry points for identification of mouse models. For example, all Centers participate in the archiving and distribution of knockout mouse models generated by the NIH Knockout Mouse Production and Phenotyping (KOMP2) Project (https://commonfund.nih.gov/komp2) (Bradley 2012; Birling 2021). In coordination with its global partners, KOMP2 is working to produce and phenotype mouse lines of male and female mice expressing null alleles for every human orthologous gene in the mouse genome. The MMRRC Program is now the primary archive and distribution repository for all KOMP2 products, including mice, germplasm, ES cells, targeting vectors, and tissue samples. Investigators seek out these popular knockout mouse lines available at a nominal cost, which is much simpler and faster than making them again in their own laboratories.

In addition, the MMRRC Program actively integrates with resources and providers of other animal species to promote scientific advances benefiting human health. For example, the MMRRC Program shares its mouse phenotype and genotype data with the MONARCH Initiative (McMurry 2016) to enable semantically integrated computational analysis across mouse and other research animal species that improves understanding the pathophysiology and genetics of human disease. These activities are essential to maximize the application of knowledge gained from studying mouse models to inform and improve human health.

## Technology transfer

The MMRRC Program has developed online Conditions of Use (COU) and Material Transfer Agreement (MTA) forms that are used for transferring mouse stocks into and out of the Centers. These documents have been developed after input from the community, with the Center’s host institutional officials, and with NIH technology transfer officers. The paperless COU and Donor MTA are designed to be time-efficient and eco-friendly, paperless forms. The Donor MTA can be printed if electronic signatures are not acceptable to a donating institution. All documents must be signed by an authorized Technology Transfer representative from the requesting or donating institution, and where applicable counter-signed by a designated Center official before the submission or request is processed. The MMRRC Program facilitates distribution of mice and materials not only to academic users but also to other non-profit and commercial, for-profit entities such as the biotechnology and biopharmaceutical sectors.

## Rigor, reproducibility, and transparency

Although reproducibility of experimental studies using mice has improved significantly from earlier (Perrin 2014), centralized mouse repository systems remain a critical and essential component to ensure the reliability of mouse models for biomedical research. The MMRRC Program is a critical component of the NIH’s initiative to optimize rigor, reproducibility and transparency in biomedical research that uses mouse models (Lloyd 2015). Centers in the MMRRC Consortium are committed to upholding the highest standards of experimental design and quality control to optimize the reproducibility of research studies using mutant mice. For example, understanding, documenting, and accurately reporting the genetic backgrounds of mouse models used in research is essential for recreating an experimental study and achieving reproducible results. Specific quality control and assurance testing processes, such as full genetic annotation and documentation of each MMRRC mouse, are in place at each Center. Also, as all Centers have committed to maintaining specific-pathogen-free vivaria and quality control measures, investigators can rely on mice obtained from the Centers to generate reproducible results. Specifically, the Centers address the NIH’s focus on rigor, reproducibility, and transparency by adhering to the following principles:

- *Scientific Premise*—the MMRRC Program provides genetically engineered mouse models for virtually every field of biomedical research ranging from neurobiology to infectious disease. These mouse models are critical to the advancement of understanding of health and disease.
- *Rigorous Experimental Design*—the MMRRC Provides authentic and key biological resources that are easily reported and described using unique identifiers (e.g., RRID numbers) to ensure full transparency so that others may obtain the same models with which to reproduce studies.
- *Consideration of Relevant Biological Variables*—models obtained from Centers in the MMRRC Consortium are genetically defined (including background strain genetics, incipient congenic strains and congenic strains) and confirmed specific pathogen free. Moreover, individual Centers have extensive expertise in troubleshooting how other biological variables, such as husbandry factors, microbiota and sex, may modulate phenotypes. By obtaining mice from the MMRRC Program, investigators eliminate the risk of genetic drift or contamination with adventitious pathogens that potentially can arise through the use of long standing in-house colonies or obtaining mice from colleagues. In addition, the MMRRC Program supports the NC3R’s ARRIVE (Animal Research: Reporting of In Vivo Experiments, https://arriveguidelines.org/) initiative to improve the reporting of research using animals (Kilkenny 2010). The MMRRC Program stands behind and supports efforts with 3R development, training, and advances and adheres to standardized reporting guidelines.
- *Authentication of Mice Using the Strain Detail Sheet*. Genetic, phenotypic, husbandry, and other scientific and research-relevant information on each mutant mouse line accepted by the MMRRC Program is documented on the MMRRC Strain Detail Sheet (SDS). Each mouse line has a unique SDS page where information provided by the donating investigator, such as specific information curated by the ICSC, relevant genotyping protocols and specifics about husbandry, care, and management can be found. In addition, users can find information on availability and prices, including fees for shipping and handling, for available product items. The online request form can be accessed directly from a mouse line’s SDS page.
- *Moving Rigor and Reproducibility Forward*. The MMRRC Program is at the forefront of developing and implementing novel broad-based strategies to optimize rigor and reproducibility of research using mouse models. Two key examples are (1) the development, refinement and application of miniMUGA for the assessment and confirmation of background strain/substrain of mutant mice and (2) investment in studies designed to understand the role of microbiota on model phenotypes and to develop tools that aid investigators in considering microbiota in model reproducibility troubleshooting and optimization. Centers in the MMRRC Consortium have adopted the highest quality control standards to ensure the fidelity and reliability of mouse models they distribute. As stated previously, all incoming lines undergo verification and characterization by a thorough physical exam by trained technicians conducting noting morphological observations, gene-specific genotyping of DNA extracted from tail snips, background strain analysis using miniMUGA genome-wide genotyping, 16s gut microbiome analysis, and pathogen surveillance via microbiological culture, PCR, and examination for parasites. Breeding performance, fertility, viability, and sexual dimorphism is noted and recorded while verifying the recoverability of newly cryopreserved germplasm. If not already available, a newly deposited mouse line is assigned a unique RRID number and listed with its full descriptive characteristics, use protocols, and available formats in the online catalog, linked to scientific references and associated databases. Upon request, a Center will attempt to recapitulate research-relevant phenotypes indicated by the submitting investigator, and importantly reveal any new or previously unknown phenotypes. When requested as live mice, strains are rederived and maintained in dedicated vivaria with strict pathogen control systems in place to prevent contamination by adventitious pathogens.

## Disaster planning to preserve and protect from catastrophic loss

Considering the steep investment over time, the scientific and unique value of the resource, and operating principles that minimize duplicative effort and redundant spending to maintain mutant mouse lines for distribution at more than one site, Centers in the MMRRC Consortium have made strategic investments to minimize losses in the event of unforeseen disasters, such as loss of power, fire, flooding, or data breach. For example, Centers split their frozen archive between different tanks and geographic sites, including at the National Animal Germplasm Program (NAGP) which is a part of the National Laboratory for Genetic Resources Preservation (NLGRP) located in Fort Collins, Colorado, USA (Kaplan 2016). Electrical power supplying the vivaria, storage tanks, and data servers are provided full 100% backup using dedicated electrical generators. Fire suppression systems are in place and monitored 24/7/365 by host institution security services, and systems are regularly tested to verify functionality. All data servers are monitored by online cloud services and monthly backups (e.g., tape) are made and stored at distant secure sites for complete data restoration if needed. These and other security practices ensure the preservation of the resource and shared between all Centers in the MMRRC Consortium.

## Resource sharing plan

The MMRRC Program was also established as a means to implement the NIH Sharing Plans for Resources and Data. Its prime directive is to encourage submission and deposition of unique mutant mouse resources developed through NIH-sponsored research into the MMRRC Program from where they are made readily available to scientists qualified and approved by their institutions to use mice for research purposes. The ICSC makes all mouse resources and genotype, health, husbandry information, phenotype, and other relevant data (including meta-analysis data) available on the SDS. Submitted data are confirmed with relevant data and terminology standards. All data are made publicly available after curation, assignment of an RRID number, and listing the strain in the MMRRC online catalog, usually within 30 days of receipt of information and mice from the submitting investigator. The ICSC provides investigators with standard language to include in publications that identify where mutant mice and data are available, how to access data on the MMRRC Program website (www.mmrrc.org), and how to acknowledge the MMRRC Program and NIH funding source.

## The future of the MMRRC program

The MMRRC Program will continue its active leadership and engagement as the leading public national mouse repository system in the USA. This includes continuing emphasis on providing research scientists with the most appropriate, valid, and reliable mutant mouse models and services using next-generation technologies (e.g., CRISPR) for well-justified, scientifically sound, and reproducible research.
